# Why and When You Should Avoid Using z-scores in Graphs Displaying Profile or Group Differences

**DOI:** 10.17505/jpor.2025.28091

**Published:** 2025-06-28

**Authors:** Julia Moeller

**Affiliations:** Leipzig University and University of Erfurt, Germany

**Keywords:** *z*-standardization, profiles, group comparisons, cluster analysis, misinterpretations

## Abstract

Many person-oriented studies use *z*-standardized scores before conducting cluster analyses and/or before displaying group differences. This article summarizes reasons why *z-* standardized scores can often be problematic and misleading in person-oriented methods. The article shows examples illustrating why and how the use of *z*-scores in group classification and comparisons can be misleading, and proposes less problematic methods. Reasons why *z*-standardized scores should be avoided when classifying or displaying differences between clusters, profiles, and other groups are:

1. The ratio of the difference between two groups is distorted in *z*-scores.
2. The ratio of the difference between two variables is distorted in *z*-scores.
3. Information about item endorsement and item rejection is lost.
4. The psychological meaning of a given *z*-score does not compare across samples and variables.
5. Group assignments can be misleading if *z*-scores are used to assign individuals to groups.
6. The group size and group frequency may be affected if *z*-scores instead of raw scores are used to assign individuals to groups.
7. Group differences in further outcome variables can change if *z*-scores instead of raw scores are used to assign individuals to groups.
8. Alternative normalization techniques perform better than *z*-standardization in cluster analyses.
9. *z*-standardization relies on homogeneity assumptions, including unimodality, but distributions analysed in person-oriented research are often multimodal.
10. Person-oriented methods typically examine within-person patterns to answer research questions about within-person phenomena, whereas *z*-standardization typically refers to between-person variation, which creates a logical mismatch between theory and method.

Alternatives to using *z*-scores in graphs displaying profiles and group differences are using raw scores or using scale transformations that use the range, not the standard deviation in the normalization.

## Introduction

*Z*-standardization is a linear transformation that transforms raw scores into z-scores. *Z*-scores are widely used to compare groups (Dere et al., 2014) and profiles (e.g., Bautista et al., 2016; Kahn et al., 2013). Moreover, public health organizations, including the World Health Organization and the US Center for Disease Control, often recommend the use of *z*-scores to relate individuals’ scores in health indices (e.g., Body Mass Index) to their reference population (e.g., de Onis et al, 1997; Ogden et al., 2002). In Psychology, *z*-standardization is probably the most frequently used approach, despite numerous alternative standardization methods.^1^ In person-oriented methods, *z*-scores are used for instance in various steps of cluster or latent profile analyses: In a first step before conducting the analysis, and in a second step after conducting the analysis to display differences between the resulting clusters or profiles in graphs (e.g., Cenkner et al., 2024; Edelsbrunner et al., 2025; Martinson et al., 2011; Nylund et al., 2007; Peng et al., 2020).

Unbeknownst to many authors, *z*-standardization can cause misinterpretations of research findings (e.g., Berkey & Colditz, 2007; Moeller, 2015). This article summarizes reasons why *z*-standardized scores should be avoided both before conducting cluster or latent profile analyses, and afterwards when displaying their results in graphs depicting profiles or mean score differences between groups. The article provides examples showing why using *z*-scores in group comparisons can be misleading, and concludes with proposing alternative methods that avoid such risks of misinterpretation.

## What is *z*-Standardization and Why is it Used to Compare Profiles and Groups?

*Z*-standardization is used to transform raw scores into z-scores. A raw score is *z*-transformed by subtracting the mean score of the sample from the raw score, and dividing the result by the standard deviation of the sample. In the resulting distribution of *z*-scores, a *z*-score of zero represents the sample mean, and a *z*-score of one or minus one is one standard deviation away from that sample mean score.

**Figure 1:**
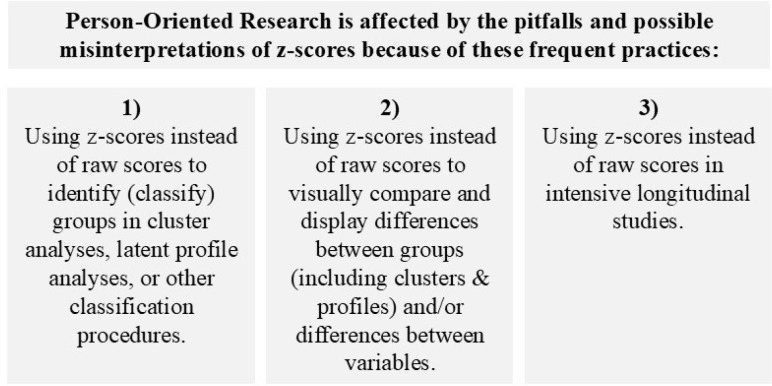
How person-oriented research is affected by pitfalls of z-standardization

In person-oriented research, *z*-standardization is used for three main purposes, depicted in Figure 1:

1. to prepare data for a subsequent cluster or latent profile analysis performed on the *z*-standardized variables,
2. to display differences between clusters, latent profiles, or other groups in graphs showing the group-specific average levels of the examined variables, and
3. in within-person analyses of longitudinal data.

Oftentimes, researchers *z*-standardize variables before conducting analyses aiming to identify unobserved groups, such as clusters or latent profiles. This practice is based on the – not necessarily correct– belief that cluster analyses or latent profile analyses perform better, that is, identify clusters or profiles better when *z*-standardized variables are used instead of raw scores. This belief is often mistaken, as we will discuss below (see Table 2 and the discussion section; see also Nylund et al., 2007).

**Table 2:** What z-standardization does, and does not, change, compared to raw scores

What z-standardization may^a^ change, compared to raw scores	What z-standardization does not change, compared to raw scores
The shapes of bi- and multivariate distributions of multiple variables (see Figure 4)	The shape of the univariate distribution of one variable
The cluster solution in a cluster analysis	The Pearson-Product-Moment correlation of two variables
The factor solution in a factor analysis	
The ratio of the mean scores belonging to two groups (see Figure 2)	The ratio of the mean score difference of
The ratio of the mean scores belonging to two variables (see Figure 3)	$\text{Ratio Differences}=\frac{\mathrm{Mean}(\mathrm{Group}1)-\mathrm{Mean}(\mathrm{Group}2)}{\mathrm{Mean}(\mathrm{Group}3)-\mathrm{Mean}(\mathrm{Group}4)}$
The univariate difference between the mean scores belonging to two groups	The univariate differences between individual scores of one variable within one person, within any given group and in the overall sample
The bi- and multivariate difference(s) between the mean scores belonging to two or more variables	
The interpretability of the psychological meaning of the response scale, including lost information about item endorsement and lost information regarding the question of which variable or which group was on average scored “higher” than the other variable or group in terms of the original response scale.	

*Note*. a = Whether or not and which of these changes occur may depend on the specific distributions, ranges of response scales, eventual outliers, mean scores, standard deviations, etc. of the variables being z-standardized.

A second use of *z*-scores in person-oriented research is to display and compare the average *z*-scores of several variables within and across unobserved groups of individuals (i.e., clusters or profiles resulting from cluster or latent profile analyses; e.g., Cenkner et al., 2024; Edelsbrunner et al., 2025; Kahn et al., 2013; Martinson et al., 2011; Nylund et al., 2007; Peng et al., 2020). In other cases, researchers compare observed groups, such as males and females, in terms of their average *z*-scores in relevant outcome variables, such as gender differences in anxiety (e.g., Dere et al., 2014).

A third use of *z*-standardization in person-oriented research transforms longitudinal data into *z*-scores by using either the within-person average, or a between-person average before applying longitudinal data analyses on the transformed scores. Why this can lead to a variety of problematic misinterpretations has been discussed elsewhere (Moeller, 2015) and is mentioned here merely because this application of *z*-scores is often combined with the above-mentioned uses of *z*-scores in person-oriented methods and group comparisons. The topic is linked to that of group comparisons because researchers planning to use *z*-standardization of longitudinal data can typically choose between several group mean scores and standard deviations as frames of reference for their *z*-transformation: The full sample’s mean score (grand mean), or the intra-individual, person-specific mean score (group mean), or the mean score of a set of repeated measures (e.g., year 1 or wave 1 in a multi-year, multi-wave data collection). The different approaches have different disadvantages and probable misunderstandings in different analyses, particularly when being used in research questions comparing the results of different observed groups (e.g., males and females) or different unobserved groups (e.g., clusters of individuals with different slopes / growth curves or trajectories over time). While the present article mainly focuses on the problems of *z*-standardization in group comparisons and leaves the problems of *z*-standardization in longitudinal studies to be discussed elsewhere (e.g., Moeller, 2015), readers should be aware of the many possible combinations of both approaches, group comparisons in longitudinal data, and the resulting risk that the problems affecting *z*-scores in either approach can accumulate and become very difficult to handle if both approaches are combined.

**Table 1:** The two steps (before and after data analysis) in which z-standardization is used in A: Analyses identifying unobserved groups and B: Analyses identifying observed groups.

	A: Analyses identifying *unobserved* groups (clusters, latent profiles, latent classes)	B: Analyses identifying *observed* groups (e.g., individuals with a high harmonious passion versus a high obsessive passion)
Step 1: uses of *z*-transformation *before* data analysis	*Rationale*: Used in the hope to better identify clusters or latent profiles statistically. If variables are measured on widely different scales (e.g., X on a scale from 0 to 10 and Y on a scale from 0 to 100), then the variable with the substantially larger response scale range is likely to show a larger variance of raw scores, which possibly influences the bi- or multivariate distances and thus clustering solution stronger than the variable with the smaller response scale range. The hope is that z-standardization avoids that variables with larger variances affect the multivariate distances stronger than variables with a smaller variance. Therefore, some scholars see it as an advantage that z-standardization can change the distances between the observations in bi- or multivariate distributions that are used to determine statistically via distance measures (e.g., Euclidean distances) which observations are similar (belonging to one cluster) or different (belonging to different clusters). *Critique*: Problems 1, 8, and 9 mentioned below. The distances in bi- or multivariate distributions do not necessarily become more trustworthy through z-standardization. There are two reasons why a variable with a larger range and variance affects a cluster solution: 1) The method artefact reflecting the fact that a variable measured on a scale from 0 to 100 has a larger range and therefore larger weight in a cluster analysis than the same variable measured on a scale from 0 to 1, and 2) The relevant information about psychological mechanisms reflected in distributions and ranges of variables, including eventually meaningful extreme groups, content-wise meaningful outliers and eventual mixture distributions. Bringing the variance of all variables to 0 through *z*-standardization removes both the method artefact, and the meaningful information reflected in the variance and range. It can lead to different cluster or profile solutions than distances calculated with raw scores of similarly scaled variables, or with ranks, or with or POMS- or POMP-transformed variables (see Table B-1). There is no sufficient theoretical or methodological reason to claim that z-score-based cluster solutions are more insightful.	*Rationale:* Used to create distinct groups of individuals, such as a group with higher *z*-scores in Variable *X* (e.g., harmonious passion) than in Variable *Y* (e.g., obsessive passion, for example, see Mageau et al., 2009; Philippe et al., 2009; Vallerand & Houlfort, 2003, for a critique, see Moeller et al., 2015). *Critique*: Problems 5, 6, 7, and 9 mentioned below.
Step 2: uses of z-transformation *after* data analysis	*Rationale*: Used to display and emphasize group differences. Differences between clusters and profiles look larger if displayed with *z*-scores rather than raw scores *Critique*: Problems 1, 2, 3, 4, 9, and 10 mentioned below. *Rationale 2*: Many researchers transform raw scores into z-scores to compare variables that were originally measured on different scales to each other. *Critique 2*: Problems 2, 3, 4, 9 and 10 mentioned below.	

One function of *z*-scores in group and profile comparisons is to bring different variables to the same metric, to make them comparable. That can be useful when inspecting profiles of variables measured on different response scales. A second reason why *z*-scores are used in group and profile comparisons is the researcher’s wish to emphasize differences between groups, which often appear larger in *z*-scores than in raw scores. Third, standardized scores are used to optimize the process of identifying unobserved groups in cluster analyses. For instance, the statistical software SPSS automatically *z*-standardize continuous variables before performing a two-step cluster analysis. Fourth, *z*-scores are used to relate individual observations to the distribution of a reference sample and the population from which that sample was drawn, for instance to say that “Otto scored 2 standard deviations below the mean score” (e.g., de Onis et al., 1997; Ogden et al., 2002). Public health organizations recommend the use of *z*-scores to relate an individual’s health indicators to a reference population and also to compare such *z*-scores across groups and over time (e.g., de Onis et al., 1997; Ogden et al., 2002).

Some of these approaches can be problematic and even achieve the opposite of what they intended for reasons summarized below.

## Reasons to Avoid *z*-scores in Group or Profile Comparisons

### Problem 1. The ratio of the difference between two groups is distorted in z-scores

When comparing groups, including clusters or individuals belonging to different profiles, it often matters how large a difference between two groups is. It may affect a study’s conclusion whether individuals in group A have on average twice as many versus four times as many children as group B, or if country A reported twice as many versus twenty times as many deaths as country B during a pandemic.

**Figure 2:**
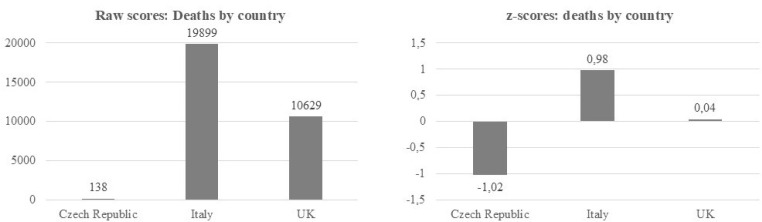
Reported deaths due to Covid-19 infections by country on April 12^th^, 8:29 p.m. (based on data from the Johns Hopkins University, 2020).

However, if groups are compared, *z*-scores may distort the relative ratios in the comparisons between groups, compared to raw scores. For example, compare the graphs for Italy and the UK in Figure 2, which show the reported deaths due to Covid-19 infections for three countries on April 12th (Johns Hopkins University, 2020). The left graph shows raw scores, the right graph the corresponding z-scores, based on the mean and standard deviation of the three countries included in the graph.

The raw scores show that Italy reported roughly twice (1.87 times) as many deaths as the UK due to Covid-19 at that date, whereas the *z*-scores suggest that Italy reported 24.50 times as many deaths as the UK. This distortion of the size of the difference between two groups can affect the effect size. The effect size *d* for instance would differ if calculated with the mean score and standard deviation for raw scores, compared to *d* being calculated with the mean and standard deviation of the *z*-scores. Maybe not many researchers would opt to calculate *d* using *z*-scores, but every reader should have the opportunity to calculate an effect size for a group difference based on the reported results of a published article, and if only *z*-scores were reported, then readers cannot calculate a realistic effect size, due to the distortions described above.

Despite such distortions in the ratios of differences between groups, some researchers purposefully use *z*-scores to emphasize the visual differences between two groups in graphs, which may look smaller or imperceptible if raw scores are displayed instead. As a rule of thumb, differences between groups, including clusters and profiles, tend to look larger in *z*-scores than in raw scores, because using *z*-scores to display group differences resembles the use of a truncated *y*-axis, which we are taught to avoid. Readers should always be given information about the theoretically possible and empirically observed ranges of a distribution, to decide for themselves whether they consider the distance between two observations, two groups, or two profiles, large or small. Graphs displaying raw scores typically display the information about the range and the theoretically possible minimum and maximum of a distribution on the *Y*-axis, unless the *Y*-axis is truncated, whereas *z*-scores obfuscate that information.

### Problem 2. The ratio of the difference between two variables is distorted in z-scores

**Figure 3:**
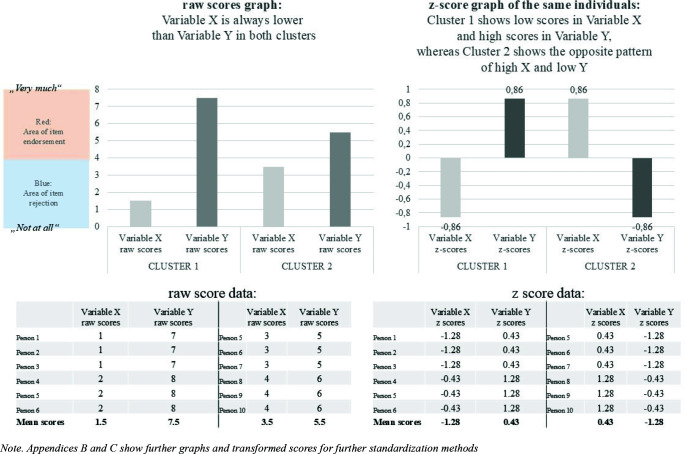
Illustrating an example in which raw-scores suggest that Variable X is generally lower than Variable Y in two groups, whereas z-scores of the same individuals suggest that Variable X is higher than Variable Y in one group. Note. Appendices B and C show further graphs and transformed scores for further standardization methods

*Z*-scores distort the ratios between the scores of two variables, compared to raw scores. This implies that the form of profiles may change dramatically and even turn completely around when *z*- scores instead of raw scores are used to display profiles. An illustration of that problem is shown in Figure 3, in which cluster 2 displays lower mean scores for Variable *X* than for Variable *Y* only when raw scores are examined, whereas that picture turns around for the same cluster 2 and the same individuals when *z*-scores are examined instead and the *z*-score-based mean score of Variable *X* becomes higher than that of Variable *Y*. Thus, the interpretation of the cluster 2 swaps from “low Variable *X*, high Variable *Y*” in terms of raw scores to “high Variable *X*, low Variable *Y*” in terms of *z*-scores.

That *z*-scores can distort the ratios between two variables, compared to raw scores, also implies that it is not possible to determine whether a score in one variable was originally larger or smaller than the score of another variable in a profile. That can have dramatic consequences for the interpretation and conclusion of a study. An illustration of that problem is shown in Figure 3, in which raw scores suggest that Variable *X* was always lower than Variable *Y* in all clusters, whereas the *z*-scores suggest that Variable *X* was higher than Variable *Y* in cluster 2. For a real empirical example of that problem, please see the research on the motivational construct of passion, which often examines harmonious passion (HP) and obsessive passion (OP), which are measured with the same response scale (1 = do not agree at all to 7 = completely agree; Vallerand et al., 2003). When individuals’ profiles of HP and OP were examined, the raw scores revealed that all profiles showed higher HP than OP, while the *z*-scores had suggested that one group showed higher OP than HP (Moeller et al., 2015).

Since many statistics textbooks emphasize the aspects that *z*-standardization does not change and omit the aspects that it does change, Table 2 gives an overview of the changes that can affect the distributions, statistical models, and interpretations relying on *z*-scores, compared to those relying on raw scores.

**Figure 4:**
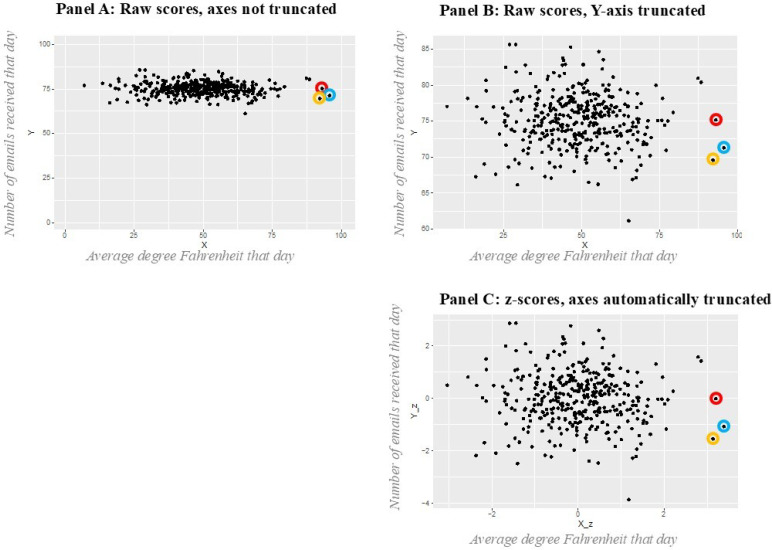
How and when z-standardization changes the bivariate distribution (Panel C), compared to raw scores (Panels A and B)

Many statistics textbooks declare that *z*-standardization does not change the distribution of a variable (e.g., Howell, 2013, p. 80). That is only partially correct, since it only applies to univariate distributions and to bi- or multivariate distributions with truncated axes. Figure 4 illustrates how and when *z*-standardization does change the bivariate distribution of two variables. For this Figure 4, two normal distributions were simulated (for the *R* code, see Appendix D). The simulation created 365 normally distributed observations for variable *X* with a mean score of 50 and a standard deviation of 15 and 365 normally distributed observations for variable *Y* with a mean score of 75 and a standard deviation of 3.5. For our thought experiment, we assume that both variables were measured on a scale from 0 to 100. As the different standard deviations already indicate, variable *X* had for whatever reason a very large range (here with a minimum of 6.901 to a maximum of 95.715) and variable *Y* had a comparably small range (here with a minimum of 61.15 to a maximum of 85.66). To give an everyday-life example illustrating why anybody would want to measure two variables with such different standard deviations on the same response scale ranging from 0 to 100, imagine *X* being the daily temperature during one year measured in Fahrenheit in the virtual city in which our virtual individual lives, and *Y* being the number of emails that virtual individual is getting each day in that year, with variable *X* being measured on a scale ranging from the historically observed minimum temperature of the virtual home city of that virtual individual (0 degree Fahrenheit) to the historically observed maximum temperature of the virtual home city of that virtual individual (100 degree Fahrenheit).

Panel A in Figure 4 shows the scatter plot of these variables *X* and *Y* with the correct, not truncated axes, both ranging from 0 to 100. Panel B in Figure 4 shows the scatter plot of these same raw scores of variables *X* and *Y*, this time with a truncated *Y*-axis, showing only the range in which empirical values for that variable *Y* were observed (60 < *Y* < 85, since our virtual person never receives less than 60 and no more than 85 emails per day in that year). Panel C in Figure 4 shows the scatter plot of the *z*-standardized scores of variables *X* and *Y*, with a truncated *Y*-axis, because graphs depicting *z*-scores typically zoom in to the section of a distribution where empirical values and variance were observed. The pictures of Panels B and C are similar, illustrating that the bivariate distribution of *z*-scores is equivalent to a bivariate distribution of raw-scores plotted on truncated axis showing only the to the section of a distribution where empirical values and variance were observed. Compared to the more correct and precise graph of Panel A, which avoids the misunderstandings of truncated axes, the bivariate distribution of the *z*-scores in Panel C looks different: For instance, consider the three highest scores of the *X*-axis encircled with the orange, red and blue circles. In Panel A, all three observations appear to be similarly far from each other in the twodimensional space; the two-dimensional distance between the observation in the red circle and the observation in the blue circle appears to be similar to the distance between the observation in the red circle and the observation in the orange circle, and similar to the distance between the observation in the blue circle and the observation in the orange circle. In contrast, these distances appear different from each other in Panels B and C, where the orange and blue observations appear to be closer to each other than each of them is to the red observation. This illustrates that in a bi- (or multi-)variate distribution, *z*-standardization can change the bi-(or multi-)dimensional distances between observations, compared to raw scores that are depicted and put in relation to their original response scale. That is the main reason for problems 2 and 3, meaning for the problem that *z*-standardization can change the distances and the ratios of two group mean scores (problem 2), and the distances and the ratios of two variable mean scores (problem 3). Thus, even though the statistics text books are correct in stating that *z*-standardization does not change the distances and ratios between observations in the univariate distribution, they should emphasize that it is nevertheless possible that a *z*-standardization will change the distances and ratios between observations in bi- or multivariate distributions (i.e., the bi- or multidimensional space). Since cluster analysis relies on exactly these bi- or multivariate distances in both identification and interpretation of differences between clusters and differences between variables, the multivariate changes to these distances created by a *z*-standardization can affect the final cluster model and the cluster interpretation in ways that resemble method artefacts and are neither theoretically, nor empirically convincing.

### Problem 3. Information about item endorsement is lost

Many studies determine the agreement or disagreement of participants with items by using a bound scale (e.g., 0 = don’t agree at all to 7 = totally agree, or 0 = does not apply at all to 10 = fully applies). In such scales, the scale midpoint is often a meaningful threshold at which the item rejection (minimal score to scale midpoint) turns into item endorsement (scale midpoint to maximal score). Whether participants agree or disagree, or whether their score exceeds a theoretically relevant cut-off is often crucial information. This information, however, is lost in *z*-scores, in which the empirical distribution instead of the original response scale becomes the frame of reference. Consequently, a *z*-score can appear to be “high” (e.g., above the mean score of *z* = 0), while representing an item rejection. Whether a *z*-score represents an item endorsement or rejection can differ for the same Variable *X* across samples, or for the same sample between variables measured on the same response scale.

An illustration of that problem is shown in Figure 3, where the raw score graph clearly shows the original demarcation at which an item rejection (scores below 5) turned into an item endorsement (scores above 5). In the *z*-score-based graph on the right side of Figure 3, it may appear as if the mean scores of Variable *X* in cluster 2 was really high, when in fact that Variable *X* had been rejected (scores below the scale midpoint of 5) by every single individual in both clusters. Likewise, the same *z*-score graph suggests that the mean scores of Variable *Y* in cluster 1 was really low, whereas in fact the average score of Variable *Y* indicated an average item endorsement (scores higher than the response scale midpoint of 5) in both clusters. This implies that for every research question for which the original response scale is a meaningful frame of reference, including all research questions asking whether a construct was experienced or not, there is a risk of misinterpretations when interpreting such *z*-score-based graphs.

### Problem 4. The psychological meaning of *z*-scores depends on the sample’s and variable’s distribution

Since *z*-scores depend on the distribution of the variable in the sample, the same *z*-score of a variable can have an entirely different psychological meaning in one sample versus another. For example, the average *z*-score of 0 of an item assessing anxiety likely indicates a normal level of anxiety in a representative sample but a clinically relevant high level of anxiety in a population of anxiety disorder patients in a closed psychiatry ward. Likewise, the same *z*-score of two variables measured on the same response scale in the same sample can have two entirely different psychological meanings, because each variable has its own frame of reference for the *z*-standardization (its own mean and standard deviation.

These sample- and variable-specific characteristics of *z*-scores make it very difficult to determine the psychological meaning of *z*-scores in graphs displaying multiple variables’ *z*-scores in one or multiple groups, and in comparisons of results across samples.

### Problem 5. People may end up in a group they do not belong to if *z*-scores are used to assign individuals to groups

Some studies not only compare *z*-scores across variables or groups, but use *z*-scores to classify individuals into distinct groups before comparing these groups’ *z*-scores in these or other variables. One risk resulting from that approach is that people may end up in a group they do not belong to according to the applied definition. For example, some studies on passion use *z*-scores of HP and OP to classify individuals into a “mainly obsessively passionate” group (*z* OP > *z* HP) and a “mainly harmoniously passionate” group (*z* OP < *z* HP; e.g., Philippe, et al., 2009). However, because of the different item difficulties of HP and OP, most individuals classified as “mainly obsessively passionate” actually show higher HP than OP, which conflicts with the definition of the group.

An illustration of this problem can be found in Figure 3: If we define individuals with a higher *z*-score in Variable *X* than in Variable *Y* as “high A, low B” (as Philippe et al., 2009 did; see Cluster 2 in Figure 3), then we classify individuals into this group (here: Cluster 2), who rated Variable *X* actually lower than Variable *Y* on the original response scale (this applies to all individuals in Cluster 2). This implies that any interpretation that includes a judgement about whether or not a score in one variable represents a higher trait expression, a higher construct level, a more intense experience, or a stronger endorsement, than the score of another variable risks being misleading if *z*-scores instead of raw scores are interpreted. At least, that affects all research questions for which the original metric (e.g., the original response scale) is a meaningful frame of reference in any way.

### Problem 6. The group size and group frequency may be affected if *z*-scores instead of raw scores are used to assign individuals to groups

Imagine that we stick with the aforementioned definition of the “mainly obsessive passionate” group as individuals with higher OP than HP, and use raw scores instead of the problematic *z*-scores to compare HP with OP. With that approach, the “mainly obsessive passionate” individuals are extremely rare, because almost no participant shows higher raw OP than HP (Moeller et al., 2015). For example, in Figure 3, the individuals with a higher score in Variable *X* than in Variable *Y* do not exist (0%), but Cluster 2 in the *z*-score based graph on the right side of Figure 3 suggests that 50% of the sample (persons 5 to 10) reported higher (*z*-)scores in Variable *X* than in Variable *Y*.

Likewise, due to possible misclassification problems described below under point 8, the size of clusters in cluster analyses may change when the variables are *z*-standardized before performing a cluster analysis on them.

### Problem 7. Group differences in further outcome variables can change if z-scores instead of raw scores are used to assign individuals to groups

Since the group composition (person-group assignment) may change if raw scores instead of *z*-scores are used to define groups (see the example of HP and OP above), a logical consequence is that group differences in outcome variables may look very different.

### Problem 8. Other scale transformations perform better than z-standardization in cluster analyses

Although many studies and statistical programs, such as SPSS, *z*-standardize continuous variables by default before using them in cluster analyses^2^, other standardization techniques using the range (max – min) instead of the standard deviation as divisor (see equation 1 below) were found to perform much better in the cluster identification in simulation studies (e.g., Everitt et al., 2001; Milligan & Cooper, 1988; Schaffer & Green, 2010). In the cited simulation studies, the true group membership is typically known, and different standardization techniques were compared in terms of their performance in identifying and distinguishing these known groups as clusters.

Based on the finding that the *z*-standardized variables lead to inefficient cluster classifications, we can deduce that the clusters identified with *z*-standardized scores may differ in size and composition from the true groups in the population that the cluster analyses aim to identify. Consequently, all problems described above under the points 5-7 may apply automatically, no matter whether the profiles are displayed in graphs using the raw scores or the *z*-scores. If the differences between the clusters are displayed in *z*-scores, then additionally the points 1-4 may apply.

Although the possible misclassifications due to *z*-scores in cluster analyses were pointed out decades ago (Milligan & Cooper, 1988), these problems remain widely unknown, as illustrated for instance in the statement “*Z* scores can be used directly in any number of analysis formats including cluster analysis“ (Cheadle et al., 2003, p. 78) and the fact that the widely-used statistical program SPSS, as well as JASP and ROPstat, still *z*-standardize continuous variables by default before various cluster analyses. Table 2 gives an overview of the inconclusive findings of several studies examining the best standardization method for cluster analysis. Possible reasons why the evidence to answer this question remains inconclusive to date, and aspects that need to be further studied in order for readers to know what standardization method to use for what kind of cluster or latent profile analysis, are discussed in the section on directions for future research below.

### Problem 9. The mean scores used in *z*-transformation require unimodality, but mixture models are often used on bi- and multimodal data

Another reason why *z*-standardization may be ill-suited for studies using latent profile analyses is their reliance on mean scores. The mean score is used twice in *z*-standardization: Once in the first step of subtracting the variables’ mean score from each observed score, and then in the second step of dividing the result by the standard deviation, which itself represents the variation of the observed values around the mean score.

Mean scores require a unimodal variable distribution in order to be meaningful measures of the central tendency of that variable (see e.g., Derrible & Ahmad, 2015; Wirtz & Nachtigall, 1998). However, as the name suggests, mixture models, such as latent profile analyses, are often used for variables that show a mixture distribution, i.e., a bi- or multimodal distribution and in fact are discouraged if all variables are bi- or multivariately normally distributed.

For example, imagine a latent profile analysis being performed on two variables that are bivariate normally distributed and positively correlated, so that individuals with relatively low values in *X* report also relatively low values in *Y*; individuals with relatively moderate values in *X* report also relatively moderate values in *Y*; and individuals with relatively high values in *X* report also relatively high values in *Y*. The latent profile analysis might conclude that there are three clusters, one consisting of individuals with low values in *X* and *Y*, one consisting of individuals with moderate values in *X* and *Y*, and one consisting of individuals with high values in *X* and *Y*. The typical reply of reviewers and colleagues to the presentation of such profiles is: A correlation would have told you the same, a clustering approach does not contribute any additional insight, compared to the – in social sciences – more common correlation-based analyses.

The unique selling point of cluster or latent profile analyses is their ability to find meaningful distinct groups of observations with distinct profiles in the studied variables that would have been overlooked with correlation-based analyses, and this unique selling point only applies to multivariate mixture distributions, meaning multivariate distributions in which at least one variable is not unimodally normally distributed. A statistical textbook would say: Correlations are the method of choice for bi- or multivariate normal distributions, but they are inappropriate for the analysis of bi- or multivariate mixture distributions, because a correlation relies on homogeneity assumptions and suggests a one-size-fits-all association for the sample, whereas mixture distributions lack such homogeneity and can consist of substantial groups of observations showing different associations than that suggested by the one-size-fits-all correlation.

Thus, cluster and latent profile analyses are typically appropriate, insightful, needed and applied to mixture distributions, and that implies that the unimodality requirements that a mean score and therefore a *z*-standardization rely on are often not present in the type of data (mixture distributions) that cluster and latent profile analyses are needed for. A researcher who believes that a mixture model might be an insightful instrument for the data at hand has likely good reasons to believe that a mean score, and by extension a *z*-transformed score, is not an insightful instrument for the data at hand.

### Problem 10. Person-oriented research typically examines within-person patterns, making it illogical to use *z*- standardization that typically refers to between-person variance

One crucial motive for applying person-oriented research is the insight that psychological theories that address within-person phenomena (e.g., patterns, mechanisms, cross-sectional and longitudinal associations) should be studied with within-person methods (e.g., within-person profiles, within-person correlations within and across time points, etc.). It has been pointed out time and again that the analysis of between-person variation does not sufficiently depict such within-person phenomena (see e.g., Molenaar, 2004; Moeller, 2021). If person-oriented research aspires to build on that insight and really focuses on the person as a whole, then it may appear illogical to go back to using between-person variance as the main frame of reference for the interpretation of the within-person patterns. *Z*-standardization typically refers to between-person variance as its frame of reference, if the between-person mean and standard deviation are used. A person-oriented researcher aiming to live up to Molenaar’s manifesto might be less interested in the question of whether a person scored comparably higher on Variable *X*, compared to other individuals, and more in the question of whether that person scored high on Variable *X* according to that person’s own cognitive understanding of that variable.

#### The core of the problem: *z*-transformation turns ratio scales into interval scales without many researchers being aware of this

Abstractly speaking, many of the misinterpretations of *z*-scores in group or profile comparisons and longitudinal analyses stem from the fact that *z*-standardized scores are interval scaled, but they are not ratio scaled. This means that it cannot be said that a *z*-score of two represents double as much of anything as a *z*-score of 1, nor does it represent half as much of anything. For instance, in terms of the absolute metric (i.e., the original metric used to measure the construct before the *z*-transformation), a *z*-score of two does not represent double the amount of the trait or construct that was measured, compared to a *z*-score of one.

Since the reference to the original metric is lost in the process of *z*-standardizing the scores, we also lose the information about ratios between two raw scores (or trait levels) measured by the scores. Some readers may argue that yes, you lose the reference to the original metric, but you gain the reference to the variable’s distribution in that sample. While that is correct, any useful information about ratios between two measures remains lost: While *z*-standardization does make every score relative to the mean score of the

distribution of its variable in the sample, that relative information also cannot be interpreted in terms of ratios: A *z*-score of 2 is twice as many standard deviations away from the mean score, but not twice as many individuals away from the mean score, since in a normal standard distribution, there are 34.1% of individuals between the mean score and the first standard deviation, but 47.7% of individuals between the mean score and the second standard deviation.

The ratio between two standard deviations (e.g., 1 *SD* compared to 2 *SD*) neither translates into the same ratio in terms of the measured construct, nor into the same ratio in terms of how many individuals away from the mean score a person with that *z*-score is. Ratios between *z*-scores lack theoretical and practical meaning. This is particularly problematic when *z*-standardization is performed on measures that originally exist on a ratio scale that has a meaningful zero point. In this case, the original metric includes meaningful information about ratios (e.g., two children are double as many as one child; four solutions created in a fluency task of a creativity test are half as many as eight solutions; see e.g., Cotter et al., 2020) and the *z*-standardization loses that information, often without the researcher being aware of that fact.

I have had discussions with colleagues who told me that the problems that I describe regarding *z*-scores may be relevant to research using LIKERT-like bound scales (e.g., response scales from 0 = not at all to 10 = very much), but that they find *z*-standardization the best and most meaningful transformation to relate unbound scales to the reference of their sample distribution (e.g., the number of children, the number of correct answers in fluency and originality ratings in creativity tests; see Cotter et al., 2020; or knowledge and competency levels in other performance tasks, see Edelsbrunner et al., 2025). At least in the discussions that I have had, the rationale of researchers arguing for *z*-standardization of unbound scales has been that the unbound scale is perceived as meaningless and that *z*-standardization adds meaningful information about whether a score is high or low in terms of the distribution. That rationale has been the main reason why the researchers that I have had discussions with opt against the available scale transformations that do not relate the transformed scores to the distribution’s mean score. The downsides of that rationale and strategy are discussed in rebuke 4 in Table 5. Most unbound scales have a natural zero point and thus are ratio scales. *Z*-standardization loses that information, whereas the POMS transformation proposed below maintains that information about ratios (see Table 4), also brings all variables to the same, intuitively understandable metric, and is easy for everyone to interpret and to compare across samples, variables, and groups.

**Table 4:** Scale transformations and their respective advantages and disadvantages for person-oriented research (for all formulas, see Appendix A)

	z-standardization	Rank (x)	Percentile Rank (x)	POMS; POMP; Min-Max	“Improve d min-max scaling”	Decimal scalin g	logarithm & generalized-log transformations	transforming the original scales by fusing extreme values on the long tail of a very skewed distribution
***Disadvantages***								
1)loses information about ratios, turns ratio scale into interval scale	yes	yes	yes	no	yes	no	yes	in part, for the fused values
2) loses information about item endorsement	yes	yes	yes	not necessaril y	yes	no	yes	possibly in part, for the fused values
3) requires unimodality to be meaningful and statistically correct	yes	no	no	no	yes	no	no	no
4) loses information about differences between variables and groups in item difficulties and mean scores	yes	yes	yes	no	yes	yes	yes	in part: removed outliers do not bias the mean score
5) Sensitive to outliers	yes	not much	not much	yes	not much	no	downplays large but exaggerates small outliers	no
***Advantages***								
1) brings variables measured on different metrics/scales to the same metric								
2) adds the information how each observation related to the variable’s distribution	yes	no	yes	yes	yes	yes	yes	no
3) Makes a non-normal distribution look more normal^a^	Yes, by anchoring it on mean score and standard deviation	Yes, by assigning each score its rank in the distributio n	Yes, by assigning each score its rank in the distributio n	no	Yes, by anchoring it on mean score and standard deviation	no	no	no
4) Is intuitive to understand at a glance in a graph	no	no	no	no	no	no	yes	A little bit, by cutting the long tail of a very skewed distribution
5) Is widely known and used in social science and person-oriented research	People think so, but it is prone to misunderstandin gs	Arguably, yes	Arguably, yes	yes	no	yes	no	Yes, but whether or not it makes sense to exclude or fuse outliers depends on data and research question
6) Recommended as best standardization method in the sophisticated simulation study by Tanioka & Yadohisa (2012)	yes	yes	yes	no	no	no	no	no

*Note*. POMS = Proportion Of Maximum Scaling; POMP = Percent Of Maximum Possible

All formulas for all transformations are shown in Appendix A. ^a^ = Whether it really is an advantage to make a non-normal distribution look more normal through standardization can be controversially debated, see Rebuke 6) in Table 4

**Table 5:** Frequent arguments brought forward in favor of z-standardization and their rebukes

Argument brought forward in favor of z-standardization	Counter argument
*Argument 1)* I have a graph depicting cluster or profile differences and the differences between profiles -or the differences between different variables, look much smaller, even unperceivable, if I display raw scores instead of z-scores. So, I choose to display z-scores to emphasize the differences between the groups.	*Rebuke 1)* Emphasizing group differences by using z-scores instead of raw scores in graphs resembles using a truncated axis, because it typically means that the parts of the axis where no difference was observed are just omitted and the parts where differences were observed are relatively enhanced. That is typically not perceived as the most realistic and trustworthy form of visual data presentation. Plus, beware of the risks that profiles can turn around, and that ratios between two variables and between two profiles become untrustworthy when z-scores instead of raw scores are used. Graphs showing z-scores can suggest very different conclusions than the showing raw scores, which can be misleading in many ways. Enhancing the perception of a small group difference does not appear a sufficient justification.
*Argument 2)* z-standardization is a linear transformation. It changes the distances between observations in one variable only by the constant added to each score and the slope coefficient by which each score is multiplied. Therefore, it can be used without problems in correlation-based analyses.	*Rebuke 2)* z-standardization does not change the distances between observations in one variable and group, but it can change the ratios of mean-score differences between two variables (see Figure C-1) and the ratios of mean-score differences between two clusters (see Table B-2, lowest line) and the psychological meaning of these ratios..
*Argument 3)* z-standardization adds information (by relating each score to the sample distribution) and therefore makes the scale more informative.	*Rebuke 3)* z-standardization also loses information (e.g., about the meaning of ratios between two scores and about the psychological meaning of the original unit) and turns scales that originally were ratio scales into mere interval scales. It therefore also makes the scale less informative and since that problem is widely ignored and interpretations about ratios are being made, it is not only a loss of information, but potentially also a loss of the knowledge about what information can, and cannot, be derived from z-scores, thus endangering the trustworthiness of interpretations of z-score-based research findings.
*Argument 4)* I have an unbound scale and if I do not z-standardize my scores, the original scores will be meaningless, because nobody knows how to interpret a score as high or low.	*Rebuke 4)* Most unbound scales have a natural zero point and thus are ratio scales. If you want to make it easier for readers to judge a score as high or low, you can use a scale that brings all measures to an easy-to-interpret metric, e.g., 0 to 1 or 0 to 100, without turning your meaningful ratio scale into the less meaningful interval scale. The POMS transformation proposed in this article maintains the ratios and its metric is easy for everyone to interpret and to compare across samples, variables, and groups. Z-standardization loses that information about ratios.
*Argument 5)* I have variables measured on different scales (e.g., Variable X: teacher feeling overwhelmed on a response scale from 1 = not at all to 10 = very much and Variable Y: teaching 0 children to 40 children in a school class) and I need to make them comparable and bring them to the same metric.	*Rebuke 5)* It is correct that with different variables measured on very different metrics, it can be necessary to bring all variables to the same metric to facilitate interpretations. There are available and easy to interpret scale transformations that bring all variables to the same metric without losing information about ratios and without causing the problems described in this article. See for instance the POMS transformation described above and compare the features of available transformations in Table 3 and Appendices A, B, and C.
*Argument 6)* I need z-standardization to transform my non-normal distribution into a more normal looking one.	*Rebuke 6)* z-standardization does not change the form of the distribution, a skewed or bimodal distribution remains in that shape after z-standardization. Even if a transformation mathematically did change the form of the distribution of the numeric relatives (i.e., the numbers representing the phenomenon -which here it does not), that transformation does not change the form of the distribution of the measured phenomenon. It is that latter distribution that researchers should be interested in. We examine how real phenomena are distributed. What would be the point in first changing the distribution of the measures artificially and then describing the form of that artificially changed distribution with our favorite statistics? If a phenomenon is heterogeneous and therefore shows a mixture distribution, then researchers need to use methods that describe such heterogeneity and that can deal with mixture distributions, instead of pretending that the phenomenon is more heterogeneous than it is, just in order to apply the homogeneityrequiring nomothetic methods they like best.

## 4.Alternatives to Standardization in Group Comparisons

As alternatives to *z*-standardization, graphs displaying profiles and groups should use raw scores or alternative scale transformations that do not change the ratios between two variables or two (observed or unobserved) groups.

Raw scores have the advantage that they are easy to understand. They can easily be compared across samples and studies if the same measures and response scales are used. To understand their psychological meaning, readers do not have to translate the reported scores into the frame of reference that participants saw when responding to a question. Raw scores indicate whether a variable was endorsed by a participant, or whether the average score of a group or variable was above a relevant threshold (e.g., clinical cut-off). Disadvantages of raw scores are that they make it difficult to compare variables that were measured with different response scales, or with unbound response scales.

**Figure C-1:**
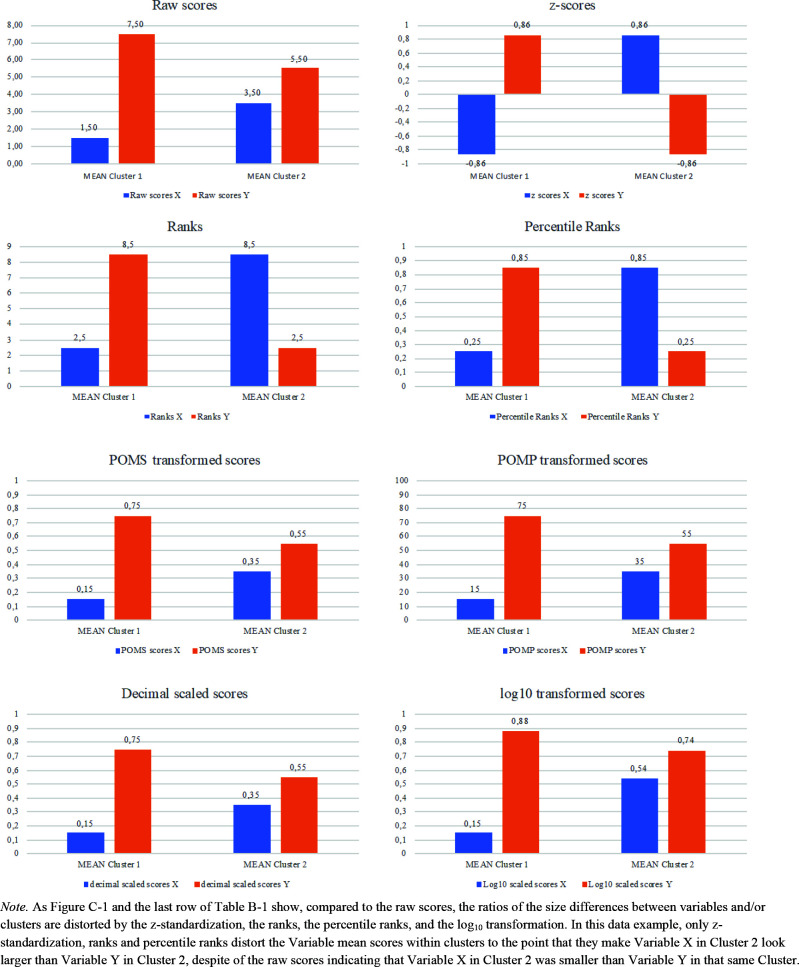
Graphs displaying the data example shown in Appendix B *Note*. As Figure C-1 and the last row of Table B-1 show, compared to the raw scores, the ratios of the size differences between variables and/or clusters are distorted by the z-standardization, the ranks, the percentile ranks, and the log10 transformation. In this data example, only z-standardization, ranks and percentile ranks distort the Variable mean scores within clusters to the point that they make Variable X in Cluster 2 look larger than Variable Y in Cluster 2, despite of the raw scores indicating that Variable X in Cluster 2 was smaller than Variable Y in that same Cluster.

There are many alternative scale transformations that can be used to standardize scores (or to normalize them, as many engineers would call the procedure). Tables 3, A-1, and B-1 and Figure C-1 show a selection of standardization procedures that have been proposed for cluster analyses and other comparisons between variables and between groups. Table 3 shows their respective advantages and disadvantages with regard to the problems discussed above. It is currently an on-going debate as to which of these transformations improve the performance of which kind of clustering algorithm under which boundary conditions. Their advantages and disadvantages are reviewed in Table 3. There is no conclusive answer yet to the question of which type of standardization performs best with which kind of clustering algorithm with which kind of data. This question is discussed in section 6, addressing directions for future research.

**Table 3:** Methods and results of different studies comparing how cluster analyses perform with different standardization methods

Reference	Types of scale transformation being compared	Data	Clustering algorithm used	Criteria used to evaluate performance of clustering algorithm	Conclusion
Tanioka & Yadohisa, (2012)	raw scores, *z*-scores, *xj* / max *xj* *xj* / (max *xj* – min *xj*) *x* / *N*$\bar{x}$ Rank (*xj*) x/(*x*^0.975^ – *x*^0.025^)	200 observations, multiple simulated datasets (Monte Carlo simulation) with varying conditions (see next cell)	Euclidean norm versus fraction norm (Minkowski norm) for kmeans clustering algorithm, compared between: Number of clusters (3 conditions), number of variables (2 conditions), variable variances (2 conditions), forms of distributions (3 conditions), error conditions (3 conditions), dissimilarities (2 conditions), levels of cluster overlap (5 conditions)	recovery results for the data standardization methods under various error conditions and dissimilarities (average value of ARI)	Rank (*xj*) “is the most effective for *k*-means clustering of all standardization methods we tested“ (Tanioka & Yadohisa, 2012, p. 66). “data standardization with the fraction norm reduces the effect of the curse of high dimensionality and gives a more effective result than data standardization with the Euclidean norm and not applying data standardization with the fraction norm.” (Tanioka & Yadohisa, 2012, p. 59)
Nogueira & Munita (2020)	*z*-score, log10, improved min–max	146 archaeological ceramic samples with each 18 elements	Hopkins statistic to determine clustering tendency. Ward & Silhouette methods to establish the number of groups. Groups generated by the k-means clustering algorithm.	internal validation indexes for compactness & separability: Dunn, Davies–Bouldin index and Calinski–Harabasz.	“the best performance was obtained with the log10 transformation. This transformation also performed well in the calculation of compactness, while the improved min–max showed better performance in terms of separability.” (Nogueira & Munita, 2020, p. 719).
Nogueira & Munita (2021)	logarithm (log), generalized-log improved minimum-maximum	data 1: 298 ceramic samples, 14 elements; data 2: 146 ceramic samples, 13 elements	Kohonen neural network	Three validation indices: Jaccard, Fowlkes Mallows and Rand	“when the cluster are close, the improved minimummaximum standardization is better than the logarithm and generalized-log. (…) when the cluster are separated, the logarithm and generalizedlog are better than the improved minimum- maximum technique.“ (Nogueira & Munita, 2021, p. 1)
Mohamad & Usman (2013)	raw scores, *z*-scores, min-max, decimal scaling	Data with 15 days and 8 Variables representing 8 of eight diseases, origin of data and meaning of scores and variables unclear	*k*-means clustering algorithm	“The sum of squares error representing distances between data points and their cluster centers and the points attached to a cluster were used to measure the clustering quality among the three different standardization methods, the smaller the value of the sum of squares error the higher the accuracy, the better the result.” (p. 3300 3301)	“standardization before clustering algorithm leads to obtain a better quality, efficient and accurate cluster result. It is also important to select a specific standardization procedure, according to the nature of the datasets for the analysis. In this analysis we proposed Z-score as the most powerful method that will give more accurate and efficient result among the three methods in K-means clustering algorithm“ (Mohamad & Usman, 2013, p. 3302).

*Annotation*. The studies are mentioned in descending order of the sophistication and quality of their methodological approach, as perceived by the author of this present article. The quality of the information provided in the study by Mohamad & Usman (2013) is considered questionable by the author of this present article.

For the comparison of clusters, latent profiles, and other groups, in graphs, this author recommends the use of the Proportion of Maximum Scaling Transformation (POMS, see Cohen et al., 1999; Gower, 1971; Little, 2013; Sneath & Sokal, 1973). The POMS is also called the Min-Max normalization (see e.g., Mohamad & Usman, 2013). It is a linear scale transformation bringing all variables to a range from 0 to 1 with the formula shown in Appendix A. POMS-transformed scores have the advantage that they provide one universal metric (from 0 to 1), so that scores can be compared across variables and samples, without any of the problems affecting *z*-scores mentioned above. The ratios in the differences between groups and between variables are unaffected by the transformation.

A disadvantage of the POMS transformation is the comparably larger effect of outliers on the min and max scores used in the POMS transformation, compared to the relatively smaller outlier effect on the mean and standard deviation used in *z*-standardization. This is likely to affect unbound scales (e.g., number of sex partners, income) stronger, because only for those the POMS formula uses the empirically observed minimum and maximum, whereas for bound scales (e.g., 1 = not at all to 5 = very much), the pre-defined min and max values are used so that the difference between outliers and non-outliers tends to be smaller than for unbound scales, depending on the phenomenon being assessed and the response options used. A way to minimize the influence of outliers on the POMS transformation for unbound scales is using more robust estimators for the sample’s min and max values, e.g., by removing outliers by trimming or winsorizing (see e.g., Wilcox, 2010). Another way to reduce the influence of outliers on a min-max transformation similar to the idea of the POMS technique is the Improved Anchored Min-Max (IAMM) Normalization Technique by Kabir et al. (2016). Outlier removal techniques to make non-normal distributions more normal can be combined with any of the standardization techniques mentioned in Tables 3, A-1, and B-1, and include the fusing of extreme values of the long tail of a very skewed distribution (Vargha & Grezsa, 2024), windsorizing or trimming (see e.g., instructions on robust statistics by Wilcox, 2010)

It should be noted that the POMS transformation strongly resembles the formula for calculating item difficulties in terms of its definition according to the Classical Test Theory (see Kelava & Moosbrugger, 2020)^3^, with the main difference that the item difficulty score according to the Classical Test Theory is multiplied by 100, making the resulting score range from 0 to 100. The same is done in the closely related percent of maximum possible (POMP) transformation, which multiplies the result of the POMS transformation by 100 (Cohen et al., 1999). Thus, calculating for each group or profile each item’s difficulty in terms of the Classical Test Theory is a further alternative to *z*-transformation and likely one that is slightly more known and easier to communicate than the POMS transformation (which, however, relies on the very same principle).

Readers interested in person-oriented research, which is a label most commonly used in social science and clinical research, should be aware of the fact that many standardization techniques are labeled differently in mathematics and engineering research, and that these fields have developed a large variety of what they call normalization methods (i.e., standardization methods in social science terminology).

## Implications for Person-Oriented Research

Many person-oriented methods use cluster or latent profile analyses and there are countless examples of studies performing these analyses on *z*-standardized indicator variables. Few studies systematically compare the results of cluster analyses conducted with raw scores versus *z*-scores. Those that do conclude that the cluster solutions differ between the two approaches (see Moeller et al., 2015; Nylund et al., 2007) and that at least for some data, there are reasons to trust the raw-score-based cluster analysis over the *z*-score-based clusters (Moeller et al., 2015).

In addition to obtaining cluster or profile solutions that may not be trustworthy if performed with *z*-transformed variables, many person-oriented studies also then display the levels of the studied variables in the different clusters in graphs showing *z*-scores (e.g., Cenkner et al., 2024; Edelsbrunner et al., 2025; Martinson et al., 2011; Peng et al., 2020). Thus, many of the interpretations of these results displayed in these graphs may be misleading, for the problems 1, 2, 3, 4, and 9 mentioned above.

Moreover, many person-oriented studies use longitudinal data in order to examine within-person patterns, and for longitudinal data there are many more pitfalls and probable misinterpretations of *z*-standardized scores, which have been discussed elsewhere (e.g., Berkey & Colditz, 2006; Moeller, 2015).

That all of these three methods – performing cluster or latent profile analyses with *z*-scores, displaying group differences in *z*-scores, and using *z*-scores in longitudinal analyses – are frequently used in person-oriented research implies that many results of such person-oriented studies risk being misinterpreted and of limited trustworthiness. At the very least, this implies that researchers using such techniques in person-oriented studies need to be aware of the risks and should aim to provide systematic comparisons between analyses performed and displayed with raw scores versus *z*-scores.

Standardization techniques using the range, such as the POMS transformation described above, avoid many of the problems that affect *z*-scores. Since there are many different methods available for standardization (or normalization, as it is often called), it is not entirely clear why the particularly problematic *z*-standardization is so frequently used in the psychological literature and in person-oriented research.

Unless researchers can make sure that the here described problems do not apply to their data, I recommend avoiding *z*-scores in the classification of unobserved groups, the displaying of observed or unobserved groups in graphs, and when working with longitudinal studies.

## Directions for Future Research

Researchers interested in person-oriented research may leave this discussion with questions such as: What standardization technique works best for the cluster or latent profile analysis or related technique that I have planned? Do the problems and misunderstandings related to *z*-standardization described in this article apply to my specific data and analyses? Do the advantages of *z*-scores outweigh the problems in my dataset and analyses? How do I navigate expectations and peer pressure in the scientific community? More specifically, how do I communicate to reviewers and readers, who are used to (mis-)interpreting *z*-scores that I chose a different, less known and less accepted, type of data standardization? For these practical questions, future research needs to provide tutorials and decision trees helping researchers pick and defend the most appropriate data standardization technique for their cluster or latent profile analysis, graph, or longitudinal analysis. So far, neither teaching materials nor published studies raise enough awareness of the problems related to *z*-scores in such analyses, nor do they provide sufficient role models and solutions showing researchers how to avoid the misinterpretations and issues related to *z*-scores discussed on this article.

An open question for future research is which kind of standardization or normalization procedure performs better, for which kind of clustering algorithm, and for which kind of dataset (varying by number of observations, clusters, overlap of clusters, cluster densities, forms of the distributions, variance of the variables, error, noise, and outliers in the data), according to which kind of quality criteria. There are very few studies that systematically compare the performance of clustering techniques across many or all of these conditions. Most available studies apply one clustering algorithm to one dataset, use one or two quality criteria to evaluate its performance, and then compare half a handful of standardization procedures in terms of how this clustering algorithm with this dataset performs with these three or so standardization procedures according to these two or so quality criteria (for examples, see e.g., Mohamad & Usman, 2013; Nogueira & Munita, 2020; 2021).

The vast majority of the available studies comparing how different standardization techniques perform in cluster analysis only examines normally distributed data. However, as I have pointed out above, person-oriented research typically uses clustering or latent profile approaches to examine mixture distributions, meaning non-normally distributed, bi- or multimodal data. I could find only one study examining for such non-normal distributions how different standardization techniques performed in clustering algorithms (Tanioka & Yadohisa, 2012). More research is needed to better understand what type of standardization is most appropriate for which form of distribution in combination with the latent profile, latent class, or cluster analyses typically performed in current person-oriented research. In line with the sophisticated example provided by Tanioka and Yadohisa (2012), such future research needs to systematically compare its results across a multitude of conditions, including different levels of cluster size, cluster number, cluster compactness and separability, noise in the data, etc.

Since most of the available studies only examine the performance of classical cluster analyses with different standardization procedures, it is rather unknown how that translates to the latent profile analyses or other latent variable approaches to clustering (e.g., latent growth curve models) that are becoming more and more popular in person-oriented research. Since cluster and latent profile analysis examined unobserved groups, it is not trivial to find useful quality criteria to decide which cluster or profile solution is the most appropriate if different analyses using differently standardized data are compared in terms of their results. One task for future studies examining the best standardization approach for latent profile and latent class models will therefore be to decide first which quality criteria to use for making the decision about what qualifies as the best solution in datasets in which group membership is not obvious.

In sum, the crucial question for future studies is not merely what standardization technique works best for cluster or latent profile analyses, but what standardization technique works best under which circumstances for which cluster or latent profile analyses. The available body of literature suggests that there will be no simple one-size-fits-all solution to this question, but it is safe to say that *z*-standardization is not the simple, unproblematic solution to this problem that many researchers currently perceive it to be.

A further avenue for future research is to determine how many person-oriented studies come to misleading conclusions because of their reliance on *z*-scores in cluster analyses, graphs, and/or longitudinal data analyses.

## Action editor

Lars-Gunnar Lundh served as action editor for this article.

## Open access

This article is distributed under the terms of the Creative Commons Attribution 4.0 International License (http://creativecommons.org/licenses/by/4.0/), which permits unrestricted use, distribution, and reproduction in any medium, provided you give appropriate credit to the original author(s) and the source, provide a link to the Creative Commons license, and indicate if changes were made.

## Appendix A Formulas of item transformations

**Table A-1:** Descriptions and formulas of different standardization techniques

Name of transformation method	Description	Formula	References
*z*-standardization	The transformed variable gets a mean score of 0 and a standard deviation of 1. Downside: Sensitive to outliers.	${\text{X}}_{\text{i}\_\text{z}}=\frac{\text{xi}-\mathrm{Mean}(x)}{\mathrm{standard}\;\mathrm{deviation}(x)}$	
Rank (x)	The numbers represent the person’s rank in relation to the sample distribution, ranging from 0 = lowest rank to the highest observed rank.		
Percentile Rank (x)	Transforms the rank into percentiles, with the sample distribution as a reference. The transformed variable gets a range from 0 to 100, with the numbers representing the person’s rank in relation to the sample distribution.	$\text{Xi\_PR}=\frac{\left(\text{Nlower}+0.5*\text{Nequal}\right)}{\text{N}}*$ 100 *legend*: Nlower = amount of scores lower than Xi Nequal = amount of scres equal to Xi N = amount of all scores	
Proportion Of Maximum Scaling (POMS); also called Min-Max normalization	The transformed variable gets a range from 0 to 1, with the numbers representing the item level in relation to the smallest and highest possible scores. Downside: Highly sensitive to outliers.	$\begin{array}{l} {\text{X}}_{\text{i}\_\text{POMS}}=\\ \frac{\text{(xi}-\mathrm{Minimum})}{(\mathrm{Maximum}-\mathrm{Minimum}} \end{array}$	Cohen et al. (1999)
Percent Of Maximum Possible (POMP)	Same formula as POMS / Min-Max, just that the result is multiplied by 100, making the range go from 0 to 100, like percentages, with the numbers representing the item level in relation to the smallest and highest possible scores. The formula is essentially similar to computing item difficulties according to the Classical Test Theory definition. Downside: Highly sensitive to outliers.	$\begin{array}{l} {\text{X}}_{\text{i}\_\text{POMP}}=\\ \frac{\text{(xi}-\mathrm{Minimum})}{(\mathrm{Maximum}-\mathrm{Minimum}}*100 \end{array}$	for item difficulty formula, see e.g., Kelava & Mossbrugger (2020)
Improved Anchored Min-Max (IAMM) Normalization Technique	Whether or not it is an improvement to mix the two reference frames (min-max of the original scale and mean score and standard deviation of the sample distribution) likely depends on data and research question. This technique was developed for the integration of multimodal (mostly psychophysiological) data.	Requires a set of steps and equations, for details, see Kabir et al. (2016).	Kabir et al. (2016)
decimal scaling standardization	Moves the decimal point of values of the variable so that the resulting score is smaller than 1.		e.g., Mohamad & Usman, 2013
logarithm & generalized-log transformations	Multiplies the raw score with a logarithm, for instance, with log(10)	X_i__log10 = log_10_(xi)	e.g., Nogueira, 2021; Nogueira & Munita, 2020
transforming the original scales by fusing extreme values on the long tail of a very skewed distribution	There are several techniques for outlier removal (e.g., trimming) or for fusing extreme values with less extreme ones (e.g., winsorizing). Whether or not that makes sense depends on data and research question and is controversially debated.	For different techniques and formulas, see e.g., Wilcox (2010)	e.g., Vargha & Grezsa (2024)

## Appendix B Data example comparing the mentioned item transformations

**Table B-1:** Example data set from Figure 1, transformed with different standardization methods

Cluster^a^	Raw scores		z scores		Ranks		Percentile Ranks		POMS scores^b^		POMP scores^b^		decimal scaled scores		log_10_ scaled scores^c^	
X	Y	X	Y	X	Y	X	Y	X	Y	X	Y	X	Y	X	Y
1	1	7	-1.28	0.43	1	7	0.1	0.7	0.1	0.7	10	70	0.1	0.7	0	0.85
1	1	7	-1.28	0.43	1	7	0.1	0.7	0.1	0.7	10	70	0.1	0.7	0	0.85
1	1	7	-1.28	0.43	1	7	0.1	0.7	0.1	0.7	10	70	0.1	0.7	0	0.85
1	2	8	-0.43	1.28	4	10	0.4	1	0.2	0.8	20	80	0.2	0.8	0.3	0.9
1	2	8	-0.43	1.28	4	10	0.4	1	0.2	0.8	20	80	0.2	0.8	0.3	0.9
1	2	8	-0.43	1.28	4	10	0.4	1	0.2	0.8	20	80	0.2	0.8	0.3	0.9
2	3	5	0.43	-1.28	7	1	0.7	0.1	0.3	0.5	30	50	0.3	0.5	0.48	0.7
2	3	5	0.43	-1.28	7	1	0.7	0.1	0.3	0.5	30	50	0.3	0.5	0.48	0.7
2	3	5	0.43	-1.28	7	1	0.7	0.1	0.3	0.5	30	50	0.3	0.5	0.48	0.7
2	4	6	1.28	-0.43	10	4	1	0.4	0.4	0.6	40	60	0.4	0.6	0.6	0.78
2	4	6	1.28	-0.43	10	4	1	0.4	0.4	0.6	40	60	0.4	0.6	0.6	0.78
2	4	6	1.28	-0.43	10	4	1	0.4	0.4	0.6	40	60	0.4	0.6	0.6	0.78
Grand mean	2.50	6.50	0.00	0.00	5.50	5.50	0.55	0.55	0.25	0.65	25.00	65.00	0.25	0.65	0.35	0.81
Grand SD	1.17	1.17	1.00	1.00	3.50	3.50	0.35	0.35	0.12	0.12	11.68	11.68	0.12	0.12	0.24	0.08
Group-Mean Cluster 1	1.50	7.50	-0.86	0.86	2.50	8.50	0.25	0.85	0.15	0.75	15.00	75.00	0.15	0.75	0.15	0.88
Group-Mean Cluster 2	3.50	5.50	0.86	-0.86	8.50	2.50	0.85	0.25	0.35	0.55	35.00	55.00	0.35	0.55	0.54	0.74
Group-SD Cluster 1	0.55	0.55	0.47	0.47	1.64	1.64	0.16	0.16	0.05	0.05	5.48	5.48	0.05	0.05	0.16	0.03
Group-SD Cluster 2	0.55	0.55	0.47	0.47	1.64	1.64	0.16	0.16	0.05	0.05	5.48	5.48	0.05	0.05	0.07	0.04
Ratio group means	**0.43**	**1.36**	-1.00	-1.00	0.29	3.40	0.29	3.40	**0.43**	**1.36**	**0.43**	**1.36**	**0.43**	**1.36**	0.28	1.18
Difference group means	-2.00	2.00	-1.71	1.71	-6.00	6.00	-0.60	0.60	-0.20	0.20	-20.00	20.00	-0.20	0.20	-0.39	0.14
Ratio group SDs	1.00	1.00	1.00	1.00	1.00	1.00	1.00	1.00	1.00	1.00	1.00	1.00	1.00	1.00	2.50	0.63
Difference group SDs	0.00	0.00	0.00	0.00	0.00	0.00	0.00	0.00	0.00	0.00	0.00	0.00	0.00	0.00	0.10	-0.02
Ratio^d^ of $\frac{\mathrm{Grand}-\mathrm{Mean}(\mathrm{Variable}\;X)}{\mathrm{Grand}-\mathrm{Mean}(\mathrm{Variable}\;Y)}$	**0.38**		Cannot be computed, no division by 0		1		1		**0.38**		**0.38**		**0.38**		0.43	
Difference of Grand-Mean(VariableX) – Grand-Mean(VariableY)	-4.00		0.00		0.00		0.00		-0.40		-40.00		-0.40		-0.46	

*Note*. ^a^ = The clusters correspond to those depicted in Figure 1. Two clusters were created deductively / artificially for the purpose of demonstrating differences between standardization methods by defining one cluster as below average values in X and above average values in Y and the other cluster as above average values in X and below average values in Y, to demonstrate the problems related to such a clustering approach, which has been used e.g., by Philippe et al. (2009) and Moneta & Csikszentmihaly (1996). ^b^ = For the purpose of this demonstration, the response scale used as frame of reference for POMS and POMP transformation ranges from 0 = not at all to 10 = very much (see Figure 1); ^c^ = rounded to the second decimal digit. This Table does not contain examples of the fused extreme values for the extreme tails of very skewed distributions mentioned in Tables 3 and B-1, because this example dataset was not skewed and because several methods of fusing or cutting outliers have been proposed in the literature. For overviews and discussions, see e.g., Wilcox (2010). ^d^ = transformations that maintained the ratio of mean scores of Cluster 2 / Cluster 1 and mean scores of Variable X / Variable Y are marked bold; transformations that distorted the ratio of mean scores of Cluster 2 / Cluster 1 and mean scores of Variable X / Variable Y compared to the raw scores are marked grey.

## Appendix D Graphs displaying the data example comparing the mentioned item transformations

*R code used to simulate the data for Figure 4*.

Please find the R code on the Open Science Framework using this link: https://osf.io/b8p6n/

## References

[cit0001] Bautista, I. J., Chirosa, I. J., Robinson, J. E., van der Tillaar, R., Chirosa, L. J., & Martínez Martín, I. (2016). A new physical performance classification system for elite handball players: Cluster analysis. *Journal of Human Kinetics*, 51, 131–142. 10.1515/hukin-2015-0177 28149376 PMC5260557

[cit0002] Berkey, C. S., & Colditz, G. A. (2007). Adiposity in adolescents: change in actual BMI works better than change in BMI *z* score for longitudinal studies. *Annals of Epidemiology*, 17(1), 44–50. 10.1016/j.annepidem.2006.07.014 17140812

[cit0003] Cenkner, D. P., Held, P., & Zalta, A. K. (2024). A latent profile analysis of moral emotions following moral transgressions. *Journal of Clinical Psychology*, 80(8), 1754–1766. 10.1002/jclp.23691 38581701

[cit0004] Cheadle, C., Vawter, M. P., Freed, W. J., & Becker, K. G. (2003). Analysis of microarray data using Z score transformation. The *Journal of Molecular Diagnostics*, 5(2), 73–81. 10.1016/S1525-1578(10)60455-2 12707371 PMC1907322

[cit0005] Cohen, P., Cohen, J., Aiken, L. S., & West, S. G. (1999). The problem of units and the circumstance for POMP (1999). *Multivariate Behavioral Research*, 34(3), 315–346. 10.1207/S15327906MBR3403_2

[cit0006] Cotter, K. N., Ivcevic, Z., & Moeller, J. (2020). Person-oriented profiles of originality and fluency in divergent thinking responses. *Journal of Research in Personality*, 86, 103941. 10.1016/j.jrp.2020.103941

[cit0007] De Onis, M., Blössner, M., & World Health Organization. (1997). WHO global database on child growth and malnutrition (No. WHO/NUT/97.4). *World Health Organization*. https://iris.who.int/handle/10665/63750 10.1093/ije/dyg099 12913022

[cit0008] Dere, E., Dahm, L., Lu, D., Hammerschmidt, K., Ju. A., Tantra, M., Kästner, A., Chowdhury, K., & Ehrenreich, H. (2014). Heterozygous Ambra1 deficiency in mice: a genetic trait with autism-like behavior restricted to the female gender. *Frontiers in Behavioral Neuroscience*, 8:181, 10.3389/fnbeh.2014.00181 24904333 PMC4032889

[cit0009] Derrible, S., & Ahmad, N. (2015). Network-based and binless frequency analyses. *PloS One*, 10(11), e0142108. 10.1371/journal.pone.0142108 26529207 PMC4631440

[cit0010] Edelsbrunner, P., Tetzlaff, L., Bach, K. M., Bichler, S., Dumas, D., Hofer, S. I., Köhler, C., Kozlova, Z., Moeller, J., Roberts, G. J., Sengewald, M.-A., & Reinhold, F. (under review). Beyond Linear Regression: Statistically Modeling Aptitude-Treatment Interactions and the Differential Effectiveness of Educational Interventions. Manuscript submitted for publication.

[cit0011] Everitt, B. S., Landau, S., & Leese, M. (2001). *Cluster analysis* (4th ed.). Arnold. https://doi.org/0.1002/9780470977811

[cit0012] Gower, J. C. (1971). A general coefficient of similarity and some of its properties. *Biometrics*, 27, 857–871. 10.2307/2528823

[cit0013] Howell, D. C. (2013). *Statistical methods for psychology* (8th ed.). Wadsworth Cengage Learning.

[cit0014] Johns Hopkins University (2020). Retrieved from https://coronavirus.jhd.edu/map/html on April 12th, 20:23 p.m.

[cit0015] Kabir W., Ahmad M. O., & Swamy M. N. S. (2016). A new anchored normalization technique for score-level fusion in multimodal biometric systems. In: *2016 IEEE international symposium on circuits and systems (ISCAS)*, (pp. 93–96). IEEE. 10.1109/access.2019.2914992

[cit0016] Kahn, R. E., Frick, P., J., Yongstrom, E. A., Kogos Yongstrom, J., Feeny, N. C., & Findling, R. L. (2013). Distinguishing primary and secondary variants of callous-unemotional traits among adolescents in a clinic-referred sample. *Psychological Assessment, 25*(3), 966–978. 10.1037/a0032880 PMC3902637 23647031

[cit0017] Kelava, A., & Moosbrugger, H. (2020). *Testtheorie und Fragebogenkonstruktion*. Springer. 10.1007/978-3-662-61532-4

[cit0018] Little, T. D. (2013). *Longitudinal structural equation modeling (Methodology in the social sciences)*. The Guilford Press.

[cit0019] Mageau, G. A., Vallerand, R. J., Charest, J., Salvy, S.-J., Lacaille, N., Bouffard, T., & Koestner, R. (2009). On the development of harmonious and obsessive passion: The role of autonomy support, activity specialization, and identification with the activity. *Journal of Personality*, 77(3), 601–646. 10.1111/j.1467-6494.2009.00559.x 20078732

[cit0020] Martinson, B. C., Vazquez Benitez, G., Patnode, C. D., Hearst, M. O., Sherwood, N. E., Parker, E. D., Sirard, J., Pasch, K. E., & Lytle, L. (2011). Obesogenic family types identified through latent profile analysis. *Annals of Behavioral Medicine*, 42(2), 210–220. 10.1007/s12160-011-9286-9 21638195 PMC3184384

[cit0021] Masyn, K.E. (2013). Latent class analysis and finite mixture modelling, in Little, T.D.(Ed.), *The Oxford Handbook of Quantitative Methods in Psychology* (pp.551–611). Oxford University Press. 10.1093/oxfordhb/9780199934898.013.0025

[cit0022] Milligan, G. W. & Cooper, M. C. (1988). A study of standardization of variables in cluster analysis. *Journal of Classification*, 5(2), 181–204. 10.1007/BF01897163

[cit0023] Moeller, J. (2015). A word on standardization in longitudinal studies: don’t. *Frontiers in Psychology*, 6(1389). 10.3389/fpsyg.2015.01389 PMC4569815 26441764

[cit0024] Moeller, J., Keiner, M., & Grassinger, R. (2015). Two sides of the same coin: Do the dual ’types’ of passion describe distinct subgroups of individuals? *Journal for Person-Oriented Research*, 1(3), 131–150. 10.17505/jpor.2015.15

[cit0025] Moeller J. (2022). Averting the Next Credibility Crisis in Psychological Science: Within-Person Methods for Personalized Diagnostics and Intervention. *Journal for Person-Oriented Research*, 7(2), 53–77. 10.17505/jpor.2021.23795 35462628 PMC8826406

[cit0026] Mohamad, I. B. & Usman, D. (2013). Standardization and Its Effects on K-Means Clustering Algorithm. *Research Journal of Applied Sciences Engineering And Technology*, 6(17), 3299–3303. 10.19026/rjaset.6.3638

[cit0027] Molenaar, P. C. M. (2004). A Manifesto on Psychology as Idiographic Science: Bringing the Person Back Into Scientific Psychology, This Time Forever. *Measurement: Interdisciplinary Research and Perspectives*, 2(4), 201–218. 10.1207/s15366359mea0204_1

[cit0028] Moneta, G., & Csikszentmihalyi, M. (1996). The effect of perceived challenges and skills on the quality of subjective experience. *Journal of Personality*, 64, 275–310. 10.1111/j.1467-6494.1996.tb00512.x 8656320

[cit0029] Nogueira, A. L., & Munita, C. S. (2020). Quantitative methods of standardization in cluster analysis: finding groups in data. *Journal of Radioanalytical and Nuclear Chemistry*, 325(3), 719–724. 10.1007/s10967-020-07186-6

[cit0030] Nylund, K., Bellmore, A., Nishina, A., & Graham, S. (2007). Subtypes, severity, and structural stability of peer victimization: What does latent class analysis say? *Child Development*, 78(6), 1706–1722. 10.1111/j.1467-8624.2007.01097.x 17988316

[cit0031] Ogden C.L., Kuczmarski R.J., Flegal K.M., Mei, Z., Guo, S., Wei, R., Grummer-Strawn, L. M., Curtin, L. R., Roche, A. F., & Johnson, C. L. (2002). Centers for Disease Control and Prevention 2000 growth charts for the United States: improvements to the 1977 National Center for Health Statistics version. *Pediatrics* 109(1), 45–60. 10.1542/peds.109.1.45 11773541

[cit0032] Peng, P., Fuchs, D., Fuchs, L. S., Cho, E., Elleman, A. M., Kearns, D. M., Patton, S., 3rd & Compton, D. L. (2020). Is “response/no response” too simple a notion for RTI frameworks? Exploring multiple response types with latent profile analysis. *Journal of Learning Disabilities*, 53(6), 454–468. 10.1177/0022219420931818 32623947 PMC7537763

[cit0033] Philippe, F. L., Vallerand, R. J., & Lavigne, G. L. (2009). Passion does make a difference in people’s lives: A look at well-being in passionate and non-passionate individuals. *Applied Psychology: Health and Well-Being, 1*, 3–22. 10.1111/j.1758-0854.2008.01003.x

[cit0034] Schaffer, C. M. & Green, P. E. (1996). An empirical comparison of variable standardization methods in cluster analysis. *Multivariate Behavioral Research*, 31(2), 149–167. 10.1207/s15327906mbr3102_1 26801454

[cit0035] Sneath, P. H. A., & Sokal, R. R. (1973). *Numerical taxonomy*. Freeman. 10.1038/193855a0 13914561

[cit0036] Tanioka, K., & Yadohisa, H. (2012). Effect of data standardization on the result of k-means clustering. In L. Fahrmeir, A. Hamerle, G. Tutz, & M. Vogel (Eds.), *Challenges at the interface of data analysis, computer science, and optimization: Proceedings of the 34th Annual Conference of the Gesellschaft für Klassifikation e. V., Karlsruhe, July 21–23*, 2010 (pp. 59–67). Springer. 10.1007/978-3-642-24466-7_7

[cit0037] Vallerand, R. J., Blanchard, C., Mageau, G. A., Koestner, R., Ratelle, C., Léonard, M., et al. (2003). Les passions de l'âme: On obsessive and harmonious passion. *Journal of Personality and Social Psychology*, 85(4), 756–767. 10.1037/0022-3514.85.4.756 14561128

[cit0038] Vallerand, R. J., & Houlfort, N. (2003). Passion at work: Toward a new conceptualization. In D. Skarlicki, S. Gilliland, & D. Steiner (Eds.), *Emerging Perspectives on Values in Organizations* (pp. 175–204). Information Age Publishing.

[cit0039] Vargha, A., & Grezsa, F. (2024). Exploring Types of Parent Attachment via the Clustering Modules of a New Free Statistical Software, ROP-R. *Journal for Person-Oriented Research*, 10(1), 1–15. 10.17505/jpor.2024.26255 38841563 PMC11149406

[cit0040] Wilcox, R. R. (2010). *Fundamentals of modern statistical methods. Substantially improving power and accuracy* (2nd ed.). Springer. 10.1007/978-1-4757-3522-2

[cit0041] Wirtz, M., & Nachtigall, C. (1998). *Deskriptive Statistik. Statistische Methoden für Psychologen* [Descriptive statistics. Statistical methods for psychologists] (2nd ed.). Juventa.

